# Conductive Polymer PEDOT:PSS-Based Platform for Embryonic Stem-Cell Differentiation

**DOI:** 10.3390/ijms23031107

**Published:** 2022-01-20

**Authors:** Eva Šafaříková, Jiří Ehlich, Stanislav Stříteský, Martin Vala, Martin Weiter, Jiří Pacherník, Lukáš Kubala, Jan Víteček

**Affiliations:** 1 Institute of Biophysics of the Czech Academy of Sciences, Královopolská 135, 612 65 Brno, Czech Republic; safarikova.eva@ibp.cz (E.Š.); kubalal@ibp.cz (L.K.); 2 Department of Experimental Biology, Faculty of Science, Masaryk University, Kamenice 5, 625 00 Brno, Czech Republic; jipa@sci.muni.cz; 3 Faculty of Chemistry, Brno University of Technology, Purkyňova 118, 612 00 Brno, Czech Republic; Jiri.Ehlich@vut.cz (J.E.); xcstritesky@fch.vut.cz (S.S.); vala@fch.vut.cz (M.V.); weiter@fch.vutbr.cz (M.W.); 4 International Clinical Research Center, St. Anne’s University Hospital Brno, Pekařská 53, 656 91 Brno, Czech Republic

**Keywords:** conductive polymer, PEDOT:PSS, screen print, embryonic stem cells, electrostimulation

## Abstract

Organic semiconductors are constantly gaining interest in regenerative medicine. Their tunable physico-chemical properties, including electrical conductivity, are very promising for the control of stem-cell differentiation. However, their use for combined material-based and electrical stimulation remains largely underexplored. Therefore, we carried out a study on whether a platform based on the conductive polymer poly(3,4-ethylenedioxythiophene):polystyrene sulfonate (PEDOT:PSS) can be beneficial to the differentiation of mouse embryonic stem cells (mESCs). The platform was prepared using the layout of a standard 24-well cell-culture plate. Polyethylene naphthalate foil served as the substrate for the preparation of interdigitated gold electrodes by physical vapor deposition. The PEDOT:PSS pattern was fabricated by precise screen printing over the gold electrodes. The PEDOT:PSS platform was able to produce higher electrical current with the pulsed-direct-current (DC) electrostimulation mode (1 Hz, 200 mV/mm, 100 ms pulse duration) compared to plain gold electrodes. There was a dominant capacitive component. In proof-of-concept experiments, mESCs were able to respond to such electrostimulation by membrane depolarization and elevation of cytosolic calcium. Further, the PEDOT:PSS platform was able to upregulate cardiomyogenesis and potentially inhibit early neurogenesis per se with minor contribution of electrostimulation. Hence, the present work highlights the large potential of PEDOT:PSS in regenerative medicine.

## 1. Introduction

Embryonic stem cells (ESCs) are pluripotent cells derived from the inner cell mass of blastocyst-stage embryos. Other types of stem cells (SCs) are isolated or induced from adult tissues. All SCs have unique regenerative abilities with a high potential for application in medicine [1,2]. A plethora of approaches to direct ESCs into a particular cell lineage has been developed for in vitro use. The differentiation protocol depends on the origin of the cells and the intended direction of SC differentiation. The methods to regulate differentiation could be divided into three groups: biological, chemical and physical [3]. Some growth factors and cytokines are able to stimulate ESCs and accelerate their differentiation. These are, for example, the TGF-beta family of proteins, insulin-like growth factor-1, leukemia inhibitory factor and cardiotrophin-1 [4]. Further, some chemicals can stimulate differentiation as well. These include dimethyl sulfoxide, 5-Azacytidin or ascorbic acid, as well as exogenous free radicals and reactive oxygen species [3,5]. The last group of stimulators is physical stimuli. This group includes mechanical forces, heat treatment and electrostimulation [3]. The latter mentioned can affect endogenous electrical fields, which are essential for maintaining cellular homeostasis and are involved in many biological events [3]. With the development of new conductive materials, electrostimulation is entering into the focus of basic as well as applied research.

Indeed, electrostimulation can be beneficial in tissue formation, tissue regeneration and wound healing, directed cell migration and alignment [6]. There has been a special interest in myocardial regeneration after heart failure and cardiac-related diseases since the mammalian heart has a very limited self-regeneration capacity [7]. In particular, cardiac regenerative medicine requires mature and well-defined cells in order to treat pathologies such as ischemic heart disease, cardiomyopathy and congenital cardiac birth defects in children [8,9]. Electrical stimulation can potentially be one way to achieve this goal [10,11]. In addition to enhanced cardiomyocyte formation, electrostimulation can result in the functional improvement of phenotypes (contractility, synchrony of beating) of formed cardiomyocytes even if prepared ex vivo [6,11,12,13].

The electrostimulation of ESCs requires a platform to support the cells and to allow the application of external electrical fields. The vast majority of such platforms was constructed using inorganic materials. Indeed, the first systems for ESCs were made from platinum or stainless-steel electrodes in direct contact with the culture medium [10]. More sophisticated systems used salt bridges to separate electrode buffers from the culture medium. Such an arrangement eliminated any adverse effect of the electrolysis of the culture medium, which stressed the cells to a high extent [14,15,16,17].

The recent extensive development of organic semiconductors has rendered them to be used in biological applications as well. Among them, the conductive polymer poly(3,4-ethylenedioxythiophene):polystyrene sulfonate (PEDOT:PSS) has attracted a great deal of attention. Although the polymer chain of PEDOT shows conjugation of double bonds, the major contribution to electrical conductivity results from its nanocrystalline structure with extensive stacking. Hence, PEDOT:PSS requires post-deposition treatment to ensure favorable electrical properties [14]. Due to its stability and biocompatibility, it has been suggested for biological applications. Indeed, short (a few hours) and mid-term (a few days) proof-of-concept applications have been implemented [15]. Long-term applications have been rather scarce despite the fact that PEDOT:PSS can maintain its performance if processed appropriately [16,17]. In connection with SCs, nanostructured PEDOT:PSS has been shown to manipulate cell adhesion. However, there is no clear general relationship between SC fate and PEDOT:PSS structure [18]. Further, PEDOT:PSS-based composite materials have been introduced in order to provide 3D niches for SCs. In connection with electrostimulation, they appear to have great potential in regenerative medicine as shown by their support of the development of neurons from neural SCs [18,19,20,21,22,23,24,25]. Other applications include osteogenic differentiation [22,26]. Importantly, some works have indicated that PEDOT:PSS-based material could promote SC differentiation by itself [21,22,27]. PEDOT:PSS was specifically used to promote cardiomyogenesis. The electrostimulation of mESCs with PEDOT:PSS-based electrodes resulted in the synchronous beating of clusters of cardiomyocytes [28]. Further, recent work of Roshanbinfar et al., 2018 [27] indicated that PEDOT:PSS-based hydrogel can promote cardiomyogenesis per se. The authors determined electrical conductivity to be the key player in the observed phenomenon. However, the use of organic semiconductors, including PEDOT:PSS, for a combined material-based and electrical stimulation to control SC differentiation, such as in the work of Yoshida et al., 2019 [28], remains largely underexplored. By applying such an approach, the potential of organic conductive polymers and electrostimulation for regenerative medicine could be maximized. 

This paper describes the construction of a PEDOT:PSS-based platform for the laboratory-scale electrostimulation of cells. The design took advantage of the precise screen printing of interdigitated PEDOT:PSS electrodes on a gold support. The platform was characterized in terms of biocompatibility and ability to produce electrical current at a pulsed-DC mode of operation. The impact of electrostimulation as well as the platform itself on mESC differentiation was determined based on lineage-specific gene and protein expressions. To our knowledge it is the first time that such a PEDOT:PSS-based design was used in a device for the electrostimulation of SCs. 

## 2. Results

### 2.1. Platforms for Differentiation of Mouse Embryonic Stem Cells

In order to determine the specific role of PEDOT:PSS during electrostimulation, two platform types were prepared in the 24-well-plate format. A platform containing only the gold electrodes (further referred to as the gold platform) served as a reference. The PEDOT:PSS platform was fabricated by screen-printing deposition of PEDOT:PSS paste onto the gold support. The procedure resulted in a 200 nm PEDOT:PSS layer that was stable for the entire cell-differentiation experiment.

The materials in direct contact with cells, i.e., the PEN foil, gold electrodes and PEDOT:PSS, showed wetting angles of 59.7 ± 1.5°, 79.4 ± 5.9° and 20.3 ± 3.5°, respectively.

Both platform types were coated with collagen IV to promote the adhesion of EBs (cell clusters prepared from mESCs). The biocompatibility was verified using mESCs. No adverse effects of the platforms were observed compared to the cell-culture plastics. Such an observation was supported by the occurrence of beating loci on both platforms at the end of the differentiation experiments, and the beating properties were comparable to the controls on the cell-culture plastics (Supplementary Figure S1). 

**Figure 1:**
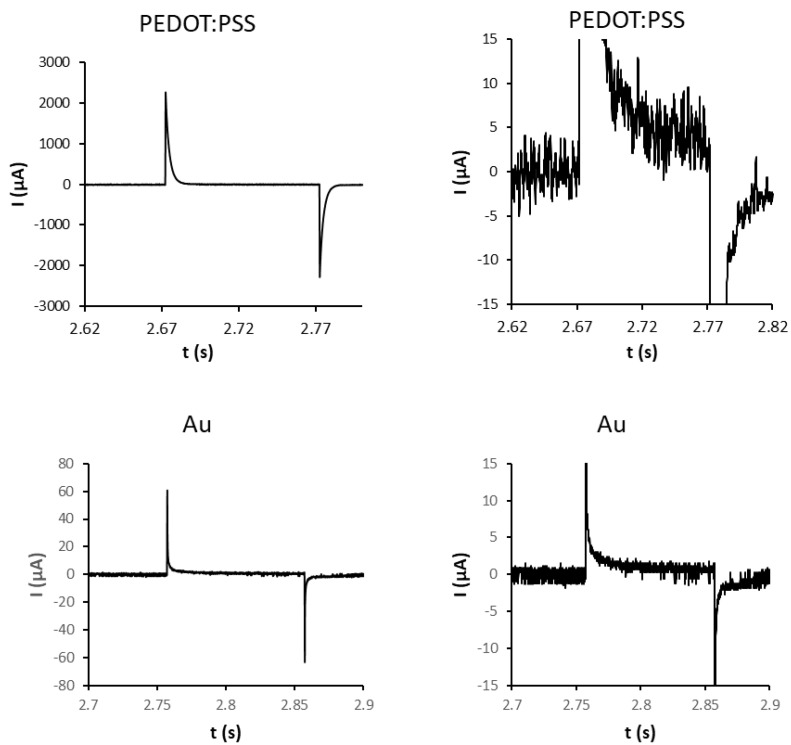
Chronoamperometric characteristics of PEDOT:PSS and gold platforms. Platform wells were loaded with PBS buffer. In two-electrode mode, the electric current was determined under pulses (1 Hz, 200 mV/mm, pulse duration 100 ms). Right column shows magnified views. Charts represent typical data.

For electrostimulation of EBs, square DC pulses (1 Hz, 200 mV/mm, pulse duration 100 ms) were chosen. In both platforms, these pulses resulted in capacitive current that reached about 2100 µA for the PEDOT:PSS platform and about 60 µA for the gold platform. The spike of capacitive current was followed by weak faradaic current that was about 5 µA for the PEDOT:PSS platform and 0.8 µA for the gold platform (Figure 1). Hence, the PEDOT:PSS platform showed about a one-order-of-magnitude-higher ratio of capacitive to faradaic current compared to the gold platform.

### 2.2. Early Response of Embryonic Stem Cells

**Figure 2:**
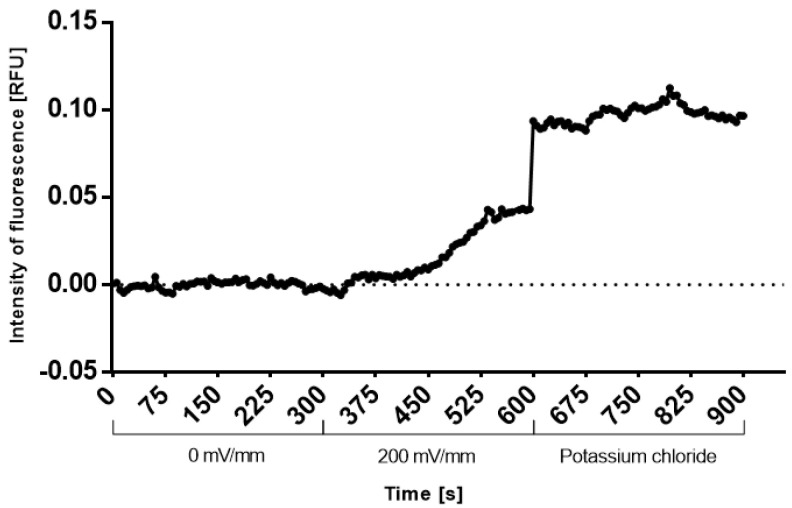
Depolarization of cytoplasmic membrane of mESCs under electrostimulation. EBs adhered to PEDOT:PSS platform wells (6 d) were loaded with voltage-sensitive probe DiBAC(4). The change in fluorescence upon stimulation with electric pulses (square wave, 1 Hz, 200 mV/mm, pulse duration 100 ms) recorded with a fluorescence microscope. Potassium chloride (40 mM) spike was used as a positive control. Records were processed in ImageJ software and normalized to the controls (0 mV/mm). The chart shows typical data from three replicates.

**Figure 3:**
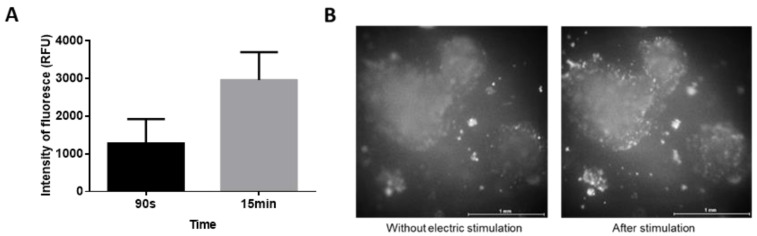
Elevation of cytosolic-calcium level under electrostimulation: EBs adhered to PEDOT:PSS platform wells (6 d) were loaded with calcium-sensitive probe Fluo-4 AM. The change in fluorescence upon stimulation with electric pulses (square wave, 1 Hz, 200 mV/mm, pulse duration 100 ms) recorded with a fluorescence microscope connected with camera. The record was processed in ImageJ software. (**A**) The chart shows typical data from three independent biological replicates. (**B**) Differences of fluorescence without electric stimulation and after stimulation for 15 min.

As the PEDOT:PSS platform showed much stronger electrical currents during the application of the 200 mV/mm square wave, its impact on early stem-cell response was estimated before the determination of changes in cell differentiation. Firstly, the membrane depolarization was checked. EBs that had adhered to PEDOT:PSS platform (6 d) were loaded with voltage probe and were electrostimulated (square DC pulses, 1 Hz, 200 mV/mm, pulse duration 100 ms). There was a rapid and sustained increase in fluorescence (Figure 2), indicating the membrane depolarization. Secondly, the possible downstream messenger of the depolarization event in cytosolic Ca^2+^ was checked by calcium-sensitive dye and a Fluo-4 AM probe. Ninety seconds of electrostimulation already showed elevated cytosolic calcium. However, the 15 min-long electrostimulation induced about three times more intense increase in cytosolic calcium (Figure 3). 

The 90 s electrostimulation resulted in no difference in beating onset compared to the controls (standard culture plastics, platform without electrostimulation). The 15 min-long electrostimulation induced the occurrence of beating loci 1–2 days earlier. Hence, the 15 min-long electrostimulation was used for further experiments.

### 2.3. Impact of Electrostimulation to Gene and Protein Expression 

To see if the mESCs were undergoing complex changes because of the platform type and/or electrostimulation, the expressions of cardiomyogenesis- and neurogenesis-marker genes were chosen. Markers of cardiomyogenesis included the mRNA level of homeobox-containing gene Nkx2.5 and genes connected with the contractile apparatus (Myh6, Myh7, Myl2 and Myl7). Further, the level of cardiac heavy myosin chains was determined. The expression of markers was followed in pre-formed EBs. 

**Figure 4:**
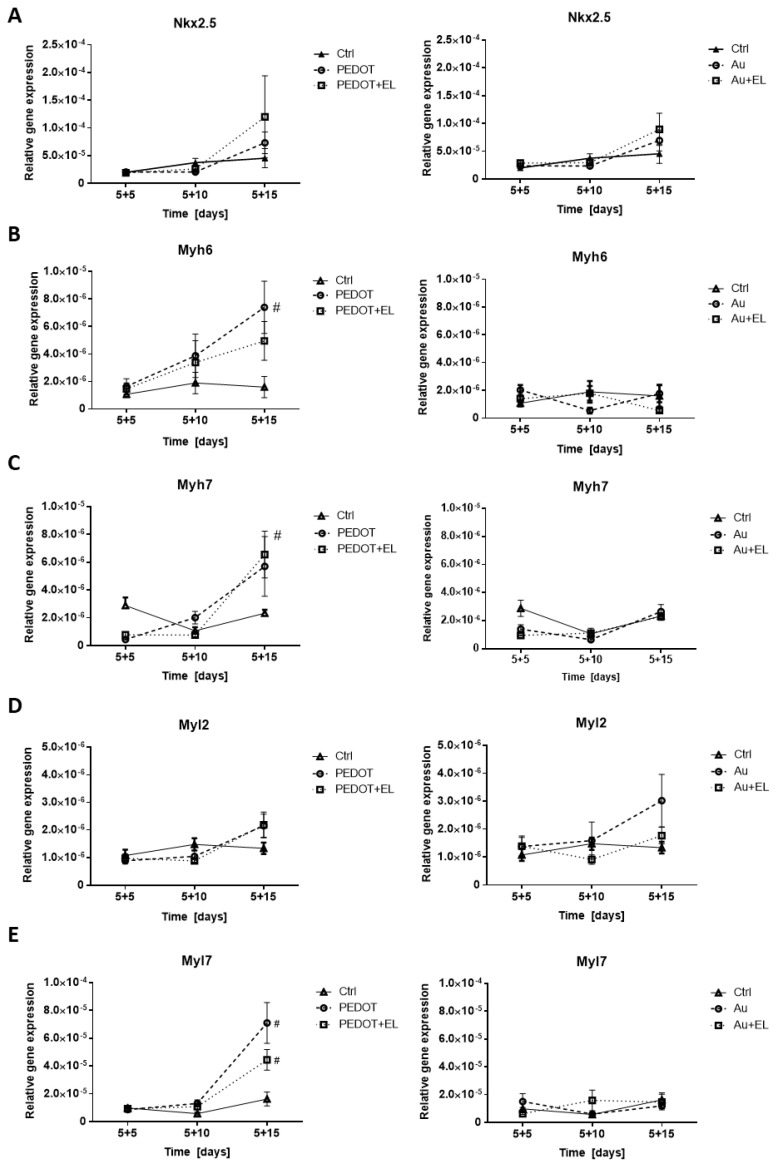
The effect of electrostimulation on expression cardiomyogenesis markers: Nkx2.5, Myh6, Myh7, Myl2, Myl7 on the PEDOT:PSS-based platform (left column) and the gold platform (right column). These genes represent markers of cardiomyocyte differentiation. The mRNA levels of NK2 transcription factor related locus 5 (Nkx2.5, (**A**)), myosin heavy chain 6 (Myh6, (**B**)), and myosin heavy chain 7 (Myh7, (**C**)), myosin light chain 2 (Myl2, (**D**)) and myosin light chain 7 (Myl7, (**E**)) were analyzed in mESC line R1 that was differentiated for 20 days. Different time points were studied (5 + 5, 5 + 10, 5 + 15 d). These time points represent individual phases of differentiation. EBs adhered to platforms were treated on day six with square electric pulses (1 Hz, 200 mV/mm, pulse duration 100 ms) for 15 min (PEDOT + EL, Au + EL). Culture plastics (Ctrl) served as the control, and a comparison to platforms without electrostimulation (PEDOT, Au) was drawn. Data are expressed as mean ± SEM (*n* ≥ 4). Differences between samples were analyzed by paired *t*-test and considered statistically significant for *p* < 0.05; they are marked with hashtags (#) for statistical significance to the control.

The data suggest a marginal trend towards the higher expression of gene Nkx2.5 on both platform materials on day 15 after electrostimulation (Figure 4A). The expressions of Myh6, Myh7, Myl2 and Myl7 shared a similar pattern and were significantly or marginally upregulated on the PEDOT:PSS platform on day 5 + 15 of the experiment. The effect of electrostimulation was indistinguishable on this platform. The electrostimulation on the gold platform as well as the gold platform without electrostimulation did not change the expression of Myh6, Myh7 and Myl7 compared to the control (Figure 4B–E). The Myl2 gene showed marginally upregulated expression on the gold platform without electrostimulation on day 5 + 15 of the experiment (Figure 4D). The ratios of the expressions of Myh6, Myh7, Myl2 and Myl7 to Nkx2.5, as well as Myh6 to Myh7 and Myl2 to Myl7, showed inconsistent or insignificant differences in comparison to the control for both platforms regardless of electrostimulation (Supplementary Figures S2–S5). Clusters of beating cardiomyocytes occurred in all variants at the end of the experiment (Supplementary Figure S1). The expression of cardiac heavy myosin chains and the occurrence of myofibrils of the contractile apparatus did not provide conclusive data (Supplementary Figure S6).

**Figure 5:**
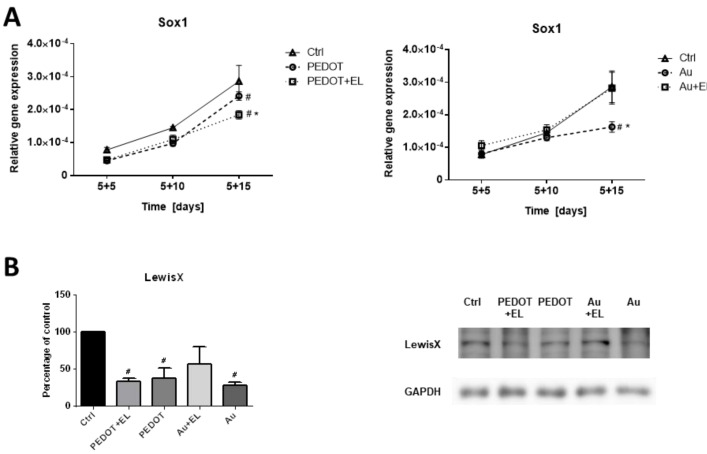
The effect of electrostimulation on expression of neural transcriptional factor Sox1 and LewisX antigen on PEDOT:PSS-based platform and platform with gold. These are markers of neural differentiation. The mRNA levels of transcription factor SOX-1 (**A**) were analyzed in mESCs line R1 which were differentiated for 20 days. Different time points were studied (5 + 5, 5 + 10, 5 + 15 d). These time points represent individual phases of differentiation. Level of antigen LewisX (**B**) was studied at the end-point (5 + 15 d). EBs adhered to platforms were treated on day 6 with square electric pulses (1 Hz, 200 mV/mm, pulse duration 100 ms) for 15 min (PEDOT + EL, Au + EL). Culture plastics (Ctrl) served as the control, and comparison to platforms without electrostimulation (PEDOT, Au) was drawn. Data are expressed as mean ± SEM (*n* ≥ 4). Differences between samples were analyzed by paired *t*-test and considered statistically significant for *p* < 0.05; they are marked with asterisks (*) for material vs. material + electrical stimulation and with hashtags (#) for statistically significance to the control.

As a marker of neurogenesis, the expression of the gene Sox1 at the mRNA level and the occurrence of LewisX antigen were chosen. Sox1 is a transcription factor specific to early neurogenesis. Compared with the control, the expression of Sox1 was marginally lowered due to the PEDOT:PSS platform regardless of electrostimulation on days 5 + 5 and 5 + 10 of the experiment. On day 5 + 15 there was statistically significant downregulation of Sox1 expression due to the PEDOT:PSS platform. The electrostimulation on this platform caused even more pronounced downregulation. Strikingly, gold did not affect the expression of Sox1 gene at early stages but induced dropdown on day 5 + 15 which was reverted by electrostimulation with this platform (Figure 5). Further, the expression of LewisX antigen (marker of neural precursor cells) was determined at the end of the experiment (day 5 + 15). LewisX antigen showed significant downregulation in cell differentiated on the PEDOT:PSS platform regardless of electrostimulation. In case of gold platform per se there was a significant decrease which was reverted by electrostimulation again (Figure 5B). 

Additionally, the expression of neurogenesis markers Nestin, Pax6, Mash1 and TuJ1 was determined at the mRNA level. The expression pattern of Nestin on the gold platform followed a very similar trend to Sox1 in the late phase of the experiment (5 + 15 d). The Pax6 and TuJ1 expressions indicated no major difference among variants. The Mash1 expression showed no major difference among variants on the PEDOT:PSS platform but its expression was elevated on gold platform without electrostimulation at the end of the experiment (5 + 15 d) (Supplementary Figure S7). 

## 3. Discussion

The use of novel materials and electrostimulation to promote and control stem-cell differentiation is constantly gaining interest as a promising tool for regenerative medicine. The selective differentiation into a specific line is of special importance. [6,11,12,13]. Further, organic conductive materials appear to be an excellent alternative to commonly used inorganic materials (e.g., gold or platinum) [19]. Therefore, we determined if a PEDOT:PSS-based platform can be beneficial to the differentiation of mESCs compared to a gold-based platform. 

The PEDOT:PSS platform was prepared using PEDOT:PSS paste deposited onto gold electrodes by screen printing. Such a method resulted in good quality and reasonably thin film, similar to the capabilities of spin coating. The major advantage was the precise deposition of PEDOT:PSS onto the supporting electrodes. Such a procedure in principle enables the production of cost-effective bio-electronic devices with submillimeter electrode size in a scalable manner. Though PEDOT:PSS in general reaches conductivity up to a few thousand Siemens, the thin layer (200 nm) in principle did not limit the overall conductivity of the platform. Importantly, the thin film was stabilized by ethylene-glycol treatment combined with heat annealing. This procedure improved the mechanical and electrical stability. Indeed, layers of PEDOT:PSS processed with ethylene glycol and thermal annealing were shown to be stable in terms of electrical conductivity for more than ten days [16]. More recent research in this field indicated that PEDOT:PSS-coated gold electrodes could be stable in physiological media for up to four months [17].

As demonstrated by the wetting angles, none of the materials in direct contact with cells (PEN foil, gold, PEDOT:PSS) showed excessively high hydrophobicity, which could prevent cell adhesion [29]. In order to yield the maximum performance from the platforms, additional protein coating was carried out. Our previous work indicated that coating of PEDOT:PSS with collagen IV can provide excellent biocompatibility to cell cultures [16,30]. Indeed, the present work supported this idea. The differentiation of ESCs into functional cardiomyocytes is a sensitive process that is only successful in very good culture conditions [31,32]. In our work, the EBs resided on the platforms for 15 days. At the end of the differentiation, we regarded them as spread EBs (with clusters of cardiomyocytes). The ability of the PEDOT:PSS platform to enable the formation of beating loci with comparable beating frequency to the culture-plastics control proved the excellent biocompatibility of the PEDOT:PSS for long-term biological applications if coated appropriately. 

Due to uncertainty about the possible combined effect of PEDOT:PSS and electrostimulation, the square DC electric pulses (1 Hz, 200 mV/mm, pulse duration 100 ms) based on the work of Hernandez et al. in 2016 [33] were applied in the two-electrode mode. In the literature, there is a range from 60 mV/mm to 750 mV/mm that is covered for various type of cells [33,34,35,36,37,38]. The voltage used in the present study corresponded to magnitude of endogenous physiological DC electrical fields that occur in animal tissues (10–200 mV/mm) [39]. Further, such electrostimulation produced a discernable biological effect, speeding up the cardiomyocyte-beating onset during the differentiation (see Section 2.2) The results clearly showed about 35 times higher capacitive current mediated by the PEDOT:PSS-covered electrodes compared with the gold electrodes. This generally corresponds with the ability of PEDOT:PSS to form high-electrical-capacity bio-interfaces [40]. The faradaic currents were about six times higher for PEDOT:PSS; hence, such material produced a one-order-of-magnitude-higher ratio of capacitive to faradaic current. The faradaic current can be described by the electrocatalytic properties of PEDOT [41,42] towards oxygen reduction [42]. The essential component of electrostimulation on both platforms was capacitive current compared to the relatively low faradaic one. However, it cannot be excluded that a minor electrocatalytic oxygen reduction could affect cells in further experiments. Additionally, a PEDOT:PSS-based platform can produce less reactive oxygen species due to the oxygen reduction per electrical current in a pulsed mode of electrostimulation. 

First, a proof-of-concept experiment with the PEDOT:PSS platform was carried out in order to determine if ESCs can produce an early response to the selected mode of electrostimulation. Our data showed that these cells could sense the stimulation as they responded with changes in membrane potential and an increase in cytosolic calcium. Such a finding is in accordance with the fact that even very short electrostimulation can have an effect on cell differentiation [33], and we point to an early mechanism dependent on membrane depolarization behind such a response. Further, the data on the cytosolic-calcium level indicate that a longer electrostimulation has the potential to elicit a more pronounced response, which could be reflected in more intense late events. Indeed, the 15 min-long electrostimulation caused beating loci to occur about 1–2 days earlier compared to culture plastics and the platform without electrostimulation. 

For differentiation, a general protocol was used. This approach did not induce any specific direction of stem-cell differentiation [43,44,45]. Hence, it provided a sensitive tool to observe if the electrostimulation of the PEDOT:PSS and gold platforms, or the platforms themselves, can modulate possible directions of differentiation. As a model system, we chose a fine balance between differentiation of ESCs to cardiomyocytes and neural cells [45]. Thus, for cardiomyogenesis the expression of transcription factor Nkx2.5 and the structural genes encoding the components of the contractile apparatus—Myh6, Myh7, Myl2, and Myl7—were chosen. The early neurogenesis was judged based on the expressions of Sox1 and Nestin in combination with the level of antigen LewisX. Additionally, expressions of Pax6, Mash1 and Tuj1 were determined to characterize later stages of neurogenesis [44].

There was a marginal trend towards elevated cardiomyogenesis due to electrostimulation regardless of the platform type, as demonstrated by the Nkx2.5 expression. This was in accordance with the earlier onset of beating after electrostimulation (see above). The expression of genes Myh6, Myh7 and Myl7 was elevated on the PEDOT:PSS platform regardless of electrostimulation. The absence of such an effect on the gold platforms strongly indicated the predominant impact of the PEDOT:PSS material to boost cardiomyogenesis. Further, it implied no effect of the gold platform itself on cardiomyogenesis. 

The expression of the contractile-apparatus genes was normalized to the level of the Nkx2.5 expression, which is relatively stable in cardiac precursor cells and cardiomyocytes. An increase in the ratio of contractile-apparatus-gene expression to the level of Nkx2.5 expression could point to a higher maturation of cells. The ratios of Myh6 to Myh7 and Myl2 to Myl7 expressions could provide information on the specific cardiomyocyte line (i.e., atrial or ventricular) [46]. The inconsistencies and absence of differences among the listed ratios indicated no enhancement in cardiomyocyte maturation and no specific direction towards a particular type of cardiomyocytes. 

The early neurogenesis was potentially reduced on the PEDOT:PSS platform. This was even more pronounced by electrostimulation on this platform, as indicated by Sox1 and the LewisX antigen. There was a potential reduction of early neurogenesis by the gold platform, as indicated by Sox1, Nestin and LewisX expressions, which could be reverted by electrostimulation. Further, the gold platform without electrostimulation showed a potential enhancement of neural maturation, as deduced from the elevated expression of Mash1. Such surprising properties of the gold platform were not documented in the literature and deserve a more detailed study beyond the scope of the present work. 

Compared to the work of Hernández and similar papers, which showed a clear shift to cardiomyogenesis, the electrostimulation using a gold-electrode system in the present study only induced a marginal shift to this direction of differentiation [10,33,36,47,48]. This was improved by the use of the PEDOT:PSS-based platform, but the effect of the material was more dominant. The combination of the improvement of cardiac differentiation by PEDOT:PSS with electrostimulation can be useful since the electrostimulation component can contribute to the functional improvement of the phenotype of formed cardiomyocytes, even if prepared ex vivo [6,11,12,13].

As demonstrated by recent works, almost all research using the PEDOT:PSS or PEDOT:PSS-based materials to electrostimulate SCs was focused on the differentiation of neural SCs or used conditions supporting neural differentiation [18,19,20,21,22,23,24,25]. In spite of this, fragments of the knowledge could be compared. Thus, a 10 min-long electrostimulation using a PEDOT:PSS-based scaffold was efficient in the upregulation of neural-cell maturation [24], confirming our finding that even a short electrostimulation of SCs can affect late events. Our findings of the effect of PEDOT:PSS per se corresponded to the literature, as PEDOT:PSS-based material has rather inhibited neurogenesis without electrostimulation [20]. 

The finding that PEDOT:PSS per se can significantly affect the ES differentiation could be hypothetically linked to the conductivity of the material or its surface properties. However, our data exclude the general electrical conductivity behind the effect of PEDOT:PSS, as it would behave in same manner as gold. In this regard, three recent works stating that PEDOT:PSS acts due to its electrical conductivity in the modulation of stem-cell differentiation should be interpreted conservatively [21,22,27]. Moyen et al. hypothesized that the effect of PEDOT:PSS could be related to its specific stiffness [18]. The coverage with collagen IV, which forms a relatively thick, 3D nonfibrillar network [49], is not supportive of the idea that stiffness is behind the action of PEDOT:PSS. On the other hand, the hydrophilicity and specific surface chemistry of PEDOT:PSS could affect the conformation of adhered proteins, which in turn could modulate the interaction with living cells [49]. 

## 4. Material and Methods

### 4.1. Electrostimulation Platform

The platform was prepared using the layout of a standard 24-well plate for cell cultures. Beneath the well plate was placed a 250 µm PEN foil (Goodlfellow Cambridge Ltd., Huntingdon, UK) as a substrate with patterned golden interdigitated electrodes. The electrodes were fabricated by physical vapor deposition, whereby the first 5 nm of NiCr alloy was evaporated as an adhesion layer that was subsequently covered by 100 nm of gold. Patterning was achieved by evaporating through a stainless-steel shadow mask. The width and spacing of the interdigitated electrodes were 500 µm. This platform was further referred to as the gold platform.

**Figure 6:**
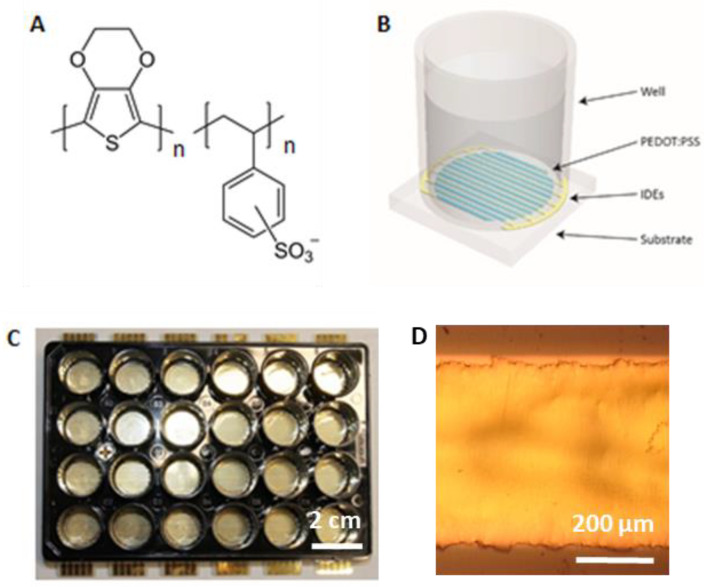
(**A**) chemical formula of PEDOT:PSS (source: https://en.wikipedia.org/wiki/PEDOT:PSS (accessed on 12 December 2021)). (**B**) Schematic view of one well in a platform used for electrostimulation. Interdigitated electrodes (IDEs) covered with PEDOT:PSS are highlighted (**C**) The electrostimulation platform in the format of 24-well plate. (**D**) Image of a section of gold IDE covered with PEDOT:PSS showing good alignment of PEDOT:PSS with gold support. Image was taken with Nikon Eclipse E200 microscope equipped with D5000 camera (Nikon Europe BV, Amsterdam, The Netherlands).

The PEDOT:PSS (formula in Figure 6A) layer was printed onto the gold electrodes using commercially available Clevios™ S V3 screen-printing pastes (Heraeus GmbH & Co. KG, Hanau, Germany). The PEDOT:PSS pattern was printed using a screen mesh count of 140 threads/cm and then functional interdigitated electrodes were created with a good alignment of PEDOT:PSS layer with the gold support. The PEDOT:PSS layer was 195 ± 21 nm thick as determined by profilometer DetakXT (Bruker, Billerica, MA, USA). The final PEDOT:PSS layer was then annealed at 140 °C for 15 min, then soaked in ethylene glycol for 15 min and annealed again at 140 °C for 15 min in order to increase the conductivity and stability of the PEDOT:PSS layer. This platform was further referred to as the PEDOT:PSS platform. The substrate and the 24-well plate were glued together using the silicone elastomer Sylgard^®^ 184 (Dow Europe GmbH, Praha, Czech Republic) and reinforced at the bottom by a 2 mm-thick acrylic sheet fixed to the well plate by screws (Figure 6B–D).

Immediately prior to any biological experiment, the platform wells were extensively washed with 70% (*v/v*) ethanol in order to remove contaminants and to sterilize surfaces. Further, the bottoms of wells were coated with murine collagen IV (cat No. 354233, BD Biosciences, Heidelberg, Germany) [30].

The chronoamperometric characterization of platforms was carried out using Potentiostat (Autolab PGSTAT 101, Metrohm Autolab B.V., Utrecht, The Netherlands). 

### 4.2. Mouse Embryonic Stem-Cell Differentiation

A general protocol of spontaneous differentiation of mESCs line R1 was used as previously described [46,50]. The cells were cultivated on gelatin-coated dishes in Dulbecco’s modified Eagle’s medium (DMEM; HyClone; Logan, UT, USA) supplemented with 15% fetal bovine serum (Gibco; Carlsbad, CA, USA), 100 IU/mL penicillin and 0.1 mg/mL streptomycin (Sigma; St. Louis, MO, USA), 1× non-essential amino acid (Gibco; Carlsbad, CA, USA), 0.05 mM β-mercaptoethanol (Fluka; Buchs, Switzerland), and 1000 U/mL of leukemia inhibitory factor (Chemicon; Temecula, CA, USA). The cells were maintained at 37 °C in humidified air supplemented with 5% CO_2_. A suspension of ES (2.5 × 10^6^ cells/mL) was seeded on the top of agarose microwells. After 24 h of formation to embryoid bodies (EBs) of uniform size (day 0), EBs were transferred to agar plates and cultivated in medium without leukemia inhibitory factor [43,51]. On day 5 (5 d), the EBs were seeded onto the stimulation platform. 

### 4.3. Stem-Cell Electrostimulation

**Figure 7:**
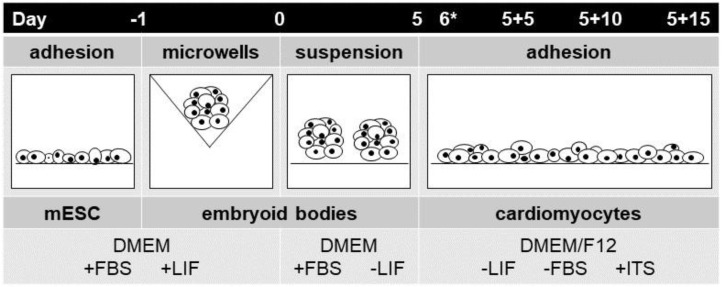
Schematic illustration of the protocol for differentiation of mESCs. For the preparation of EBs from mESCs, the silicone microwells were used. After 24 h of incubation (Day 0), the EBs were transferred onto an agar-coated dish. On day 5 (Day 5), the EBs were seeded to the 24-well stimulation platform. On day 6 (Day 6*) cells were electrostimulated.

Eight EBs on 5 d phase were transferred to each well of a platform filled with DMEM/F-12 (1:1) medium (HyClone; Logan, UT, USA) supplemented with insulin-transferrin selenium (Gibco; Carlsbad, CA, USA) and antibiotics (specification above). They were cultivated for a further 5 (5 + 5 d), 10 (5 + 10 d), 15 (5 + 15 d) days. These time points represent various stages of cardiomyocyte development. For electrostimulation it was necessary to have adherent bodies. After 24 h of adhesion, EBs were electrically stimulated. The DC electric field was 200 mV/mm at 1 Hz frequency and 100 ms pulse width. Control EBs were subjected to the same procedure but without electrical stimulation. Our stimulation protocol was inspired by Hernández et al. [33]. Cells were harvested at different time points (5 + 5 d, 5 + 10 d, 5 + 15 d), which represent various stages of cardiomyocyte development: cardiac progenitors (up to 5 days), early cardiomyocyte-like cells (up to 10 days) and beating cardiomyocyte-like cells (between 15 and 20 days) (Figure 7) [46]. 

### 4.4. Imaging of Intracellular Calcium 

Intracellular free calcium (Ca^2+^) was monitored using the fluorescent calcium indicator Fluo-4 AM (Invitrogen). Cells were incubated with Fluo-4 AM at a concentration of 5 µM for 30 min in serum-free media (DMEM/F12 1:1, HyClone; Logan, UT, USA) at 37 °C [33]. After the incubation, electrostimulation and time-lapse-image capturing (1 frame per 5 s) were started. Fluorescent images of EBs were obtained in each well using a fluorescent microscope (Axiobserver Z1, Zeiss, Germany). The fluorescence signal was visualized with a green-fluorescent-protein filter set. The image analysis was carried out using ImageJ software (version 1.47v) [52]. 

### 4.5. Depolarization Detection

To verify the sensitivity of cells to electrostimulation, the membrane-potential-sensitive probe DiBAC4(3) was used. A stock solution was prepared at a concentration of 5 mM. The stock solution was diluted in dimethyl sulfoxide to concentration 2 mM and aliquots were frozen at −20 °C. Aliquots with concentrations of 2 mM were diluted to 10 µM and cultivated in DMEM/F12 1:1 (HyClone; Logan, UT, USA) [53,54]. After 30 min of incubation, electrostimulation and time-lapse-image capturing (1 frame per 5 s) was started. Images were obtained of each well using a fluorescence microscope (Axiobserver Z1, Zeiss, Oberkochen, Germany). The fluorescence signal was visualized with a green-fluorescent-protein filter set. The image analysis was carried out using ImageJ software (version 1.47v) [52]. 

### 4.6. Gene Expression Analysis

Total RNA was extracted using the CatchGene Tissue DNA Kit (CatchGene; Taipei, Taiwan). Complementary DNA was synthesized according to the manufacturer’s instructions for the Sensiscript RT Kit (Qiagen; Germantown, MD, USA) using TurboCycler Lite thermal cycler (BlueRay Biotech, Taipei, Taiwan). Real-time quantitative PCR (RT-qPCR) reactions were performed in a LightCycler480 instrument using LightCycler480 Probe Master solutions (Roche; Basel, Switzerland). The following program was used: initial denaturation step at 95 °C for 10 min, followed by 45 cycles (95 °C for 10 s, 60 °C for 30 s, and 72 °C for 1 s) and the final cooling step at 40 °C for 1 min. Ribosomal protein L13A (RPL13A) was used as a reference gene and the expression of genes of interest was presented as 2^−Δcq^. The sequences of primers and numbers of the Universal Probe Library probes are listed in Table 1. 

### 4.7. Protein Expression Analysis

Total protein lysates were prepared from differentiated ESCs of day 5 + 15 d. Western-blot analysis, cell-sample harvesting, and preparation were performed by a standard procedure as previously presented [43]. Protein concentrations were determined using BCA Protein Assay (Pierce Biotechnology; Rockford, IL, USA) according to the manufacturer’s instructions. The following primary antibodies were used to detect antigen LewisX, which is typical of neural precursor cells (mouse monoclonal anti-LewisX/Forse1, DSHB, University of Iowa, deposited by Patterson P.H., https://dshb.biology.uiowa.edu/FORSE-1, website accessed on 20 May 2021), GPDH in 5% non-fat milk/TBS-T at 4 °C overnight. The corresponding secondary HRP-conjugated anti-rabbit 1:3000 (Sera care, Milford, MA, USA) antibody in 5% non-fat milk for 1 h at RT was employed. The amount of 10 µg of protein was loaded onto a 10% SDS-PAGE gel, transferred onto a polyvinyl difluoride membrane (Merck Millipore; Darmstadt, Germany) and blocked in 5% low-fat milk. The immunoreactivity bands were detected using an ECL detection-reagent kit (Pierce, USA) and then exposed on Amersham 680 (GE Healthcare, North Richland Hills, TX, USA). Optical densities were quantified by scanning densitometry and expressed in arbitrary units determined by ImageJ software (version 1.47v) [52].

## 5. Conclusions

In the present work, we focused on a novel and largely underexplored combined material and electrical-based stimulation of SCs. Gold- as well as PEDOT:PSS-based platforms showed excellent biocompatibility. The PEDOT:PSS platform was able to produce a higher electrical current with the pulsed-DC electrostimulation mode (1 Hz, 200 mV/mm, 100 ms pulse duration) compared to the gold platform. In proof-of-concept experiments, ESCs were able to respond to such electrostimulation by membrane depolarization and elevation of cytosolic calcium. Electrical stimuli on both platforms induced a marginal trend towards cardiomyogenesis, as deduced from the expression of the Nkx2.5 gene, whereas on the gold platform it potentially attenuated the early neurogenesis, as demonstrated by the decrease in the expression of gene Sox1 and LewisX antigen. There was a boost of cardiomyogenesis on the PEDOT:PSS platform indicated by the elevated expression of the genes encoding the contractile apparatus. However, the effect of the PEDOT:PSS platform itself was dominant. The gold platform per se did not affect cardiomyogenesis because there was no change in expression of the contractile apparatus, but it potentially reduced early neurogenesis as demonstrated by the lowered expression of Sox1 and Nestin genes and the LewisX antigen. Taken together, an effect of PEDOT:PSS platform itself to upregulate cardiomyogenesis and potentially inhibit early neurogenesis with the minor contribution of electrostimulation could be concluded. Therefore, the present work further highlights the large potential of PEDOT:PSS in regenerative medicine.

## Figures and Tables

**Table 1:** Sequence of primers used in quantitative RT-PCR.

Gene of Interest	Forward Primer 5′→3′	Reverse Primer 5′→3′	UPL Probe No.
*Rpl13a*	CATGAGGTCGGGTGGAAGTA	GCCTGTTTCCGTAACCTCAA	#25
*Nkx2.5*	GACGTAGCCTGGTGTCTCG	GTGTGGAATCCGTCGAAAGT	#53
*Myh6*	CGCATCAAGGAGCTCACC	CCTGCAGCCGCATTAAGT	#6
*Myh7*	CGCATCAAGGAGCTCACC	CTGCAGCCGCAGTAGGTT	#6
*Myl2*	CCCAGATCCAGGAGTTCAAG	CTGCAGCCGCAGTAGGTT	#95
*Myl7*	CCCATCAACTTCACCGTCTT	AACATGCGGAAGGCACTC	#7
*Sox1*	GTGACATCTGCCCCCATC	GAGGCCAGTCTGGTGTCAG	#60

## Data Availability

The data presented in this study are available on request from the corresponding author.

## Supplementary Materials

The following supporting information can be downloaded at: https://www.mdpi.com/article/10.3390/ijms23031107/s1.

## Abbreviations

embryoid bodies	(EBs)
stem cells	(SCs)
embryonic stem cells	(ESCs)
mouse embryonic stem cells	(mESCs)
poly(3:4-ethylenedioxythiophene):polystyrene sulfonate	(PEDOT:PSS)
direct current	(DC)
