# Supplementary material for: Conductive Polymer PEDOT:PSS-Based Platform for Embryonic Stem-Cell Differentiation
Source: Int J Mol Sci. 2022 Jan 20;23(3):1107. doi: 10.3390/ijms23031107 (PMC8835127; doi:10.3390/ijms23031107)
Supplement: Supplementary file 1 [file ijms-23-01107-s001.zip › ijms-1542357-supplementary.pdf]

Supplementary material

Conductive Polymer PEDOT:PSS Based Platform for Embryonic Stem Cells Differentiation

Eva Šafaříková, Jiří Ehlich, Stanislav Stříteský, Martin Vala, Martin Weiter, Jiří Pacherník, Lukáš Kubala and Jan Víteček

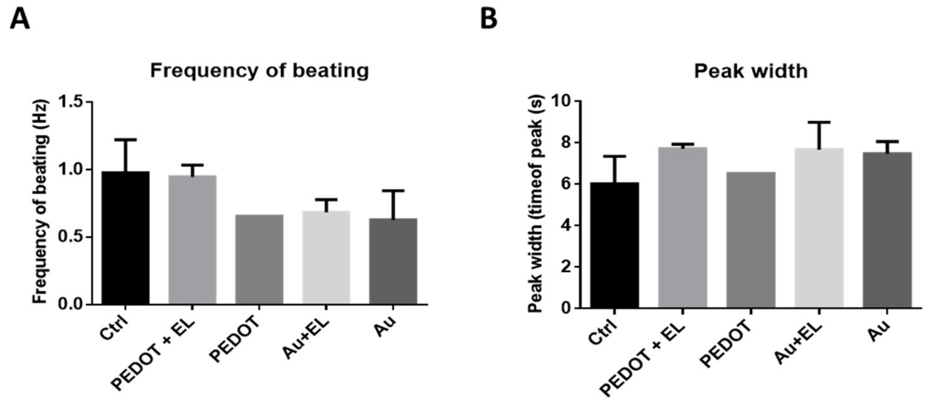

Figure S1. Analysis of beating of cardiomyocytes differentiated out of mESCs. Quantification of beating frequency (A) and duration of contraction (the peak width) (B) of beating cardiomyocytes at 5+15 day of the experiment. EBs adhered to platforms were treated on day 6 with square electric pulses (EL) (1 Hz, 200 mV/mm, pulse duration 100 ms) for 15 minutes (PEDOT+EL, Au+EL). Culture plastics (Ctrl) served as control, and comparison to platforms without electrostimulation (PEDOT, Au) was drawn. Videos were analyzed by the software CBA analyser software [1]. Data are expressed as mean ± SEM, Differences between samples were analysed by paired t-test and considered statistically significant for $p < 0.05$ (n =3, except of PEDOT: n = 1).

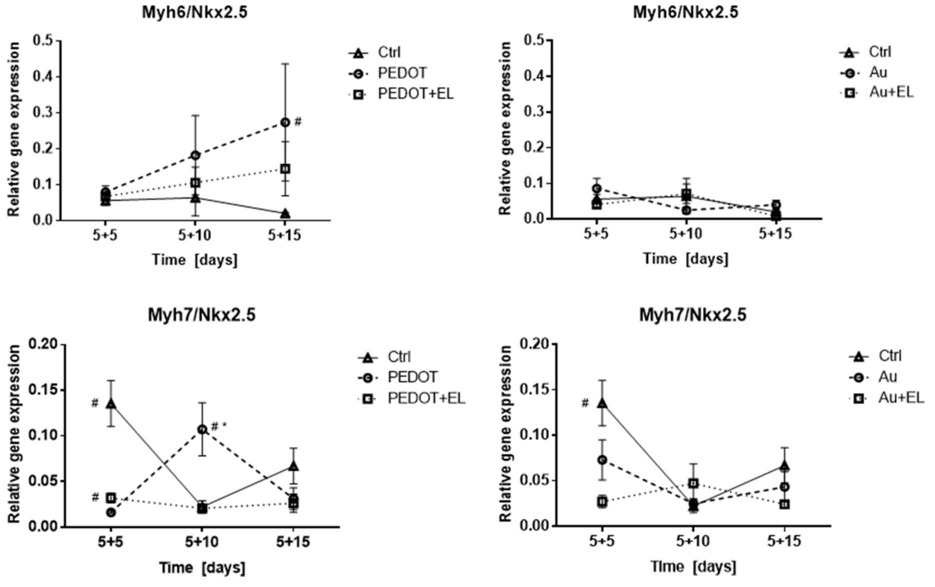

Figure S2. The ratios of Myhs expression to Nkx2.5 expression in cardiomyocytes on PEDOT:PSS based platform and platform with gold. The ratios of mRNA levels of Myh6 and Myh7 to Nkx2.5 were analyzed in mESCs line R1 which were differentiated for 20 days. Different time points were studied (5+5, 5+10,5+15d). This time point represents individual phases of differentiation. EBs adhered to platforms were treated on day 6 with square electric pulses (1 Hz, 200 mV/mm, pulse duration 100 ms) for 15 minutes (PEDOT+EL, Au+EL). Culture plastics (Ctrl) served as control, and comparison with platforms without electrostimulation (PEDOT, Au) was drawn. Data are expressed as mean ± SEM n ≥ 4). Differences between samples were analysed by paired t-test and considered statistically significant for $p < 0.05$; they are marked with asterisks for material vs. material + electrical stimulation and with hashtags for statistically significance to control.

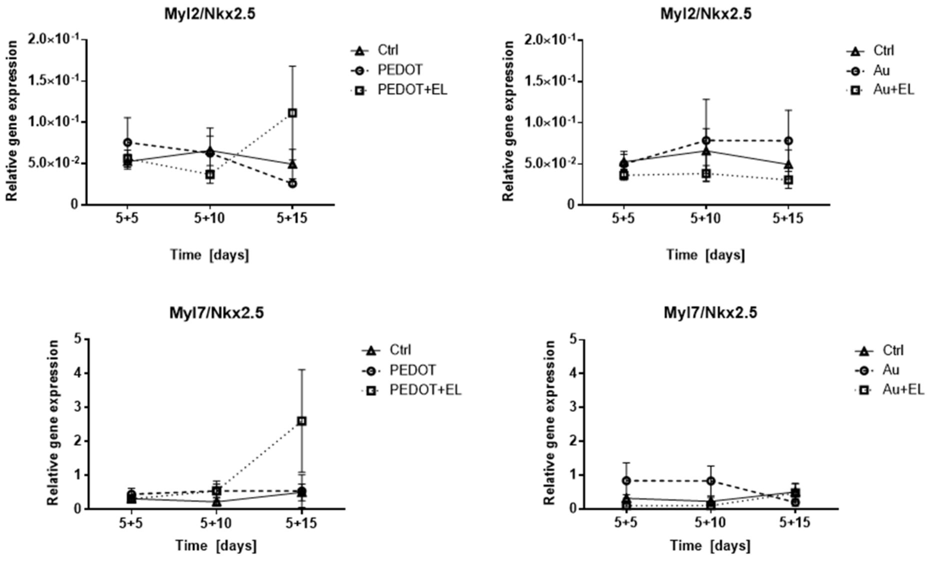

Figure S3. The ratios of Myls expression to Nkx2.5 expression in cardiomyocytes on PEDOT:PSS based platform and platform with gold. The ratios of mRNA levels of Myl2 and Myl7 to Nkx2.5 were analyzed in mESCs line R1 which were differentiated for 20 days. Different time points were studied (5+5, 5+10,5+15d). This time point represents individual phases of differentiation. EBs adhered to platforms were treated on day 6 with square electric pulses (1 Hz, 200 mV/mm, pulse duration 100 ms) for 15 minutes (PEDOT+EL, Au+EL). Culture plastics (Ctrl) served as control, and comparison with platforms without electrostimulation (PEDOT, Au) was drawn. Data are expressed as mean ± SEM n ≥ 4). Differences between samples were analysed by paired t-test and considered statistically significant for $p < 0.05$; they are marked with asterisks for material vs. material + electrical stimulation and with hashtags for statistically significance to control.

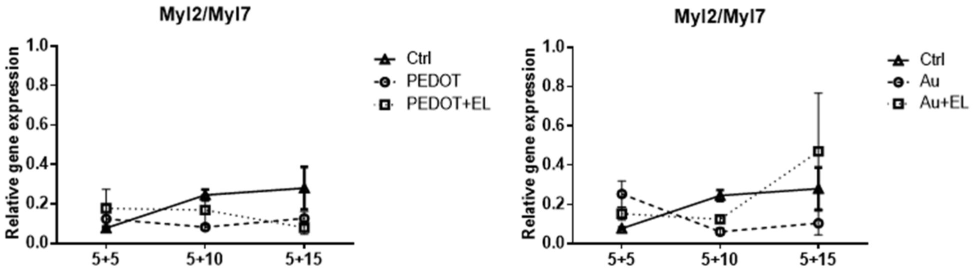

Figure S4. The ratios of Myl2 expression to Myl7 expression in cardiomyocytes on PEDOT:PSS based platform and platform with gold. The ratio of mRNA levels of Myl2 to Myl7was analyzed in mESCs line R1 which were differentiated for 20 days. Different time points were studied (5+5, 5+10,5+15d). This time point represents individual phases of differentiation. EBs adhered to platforms were

treated on day 6 with square electric pulses (1 Hz, 200 mV/mm, pulse duration 100 ms) for 15 minutes (PEDOT+EL, Au+EL). Culture plastics (Ctrl) served as control, and comparison with platforms without electrostimulation (PEDOT, Au) was drawn. Data are expressed as mean ± SEM n ≥ 4). Differences between samples were analysed by paired t-test and considered statistically significant for $p < 0.05$; they are marked with asterisks for material vs. material + electrical stimulation and with hashtags for statistically significance to control.

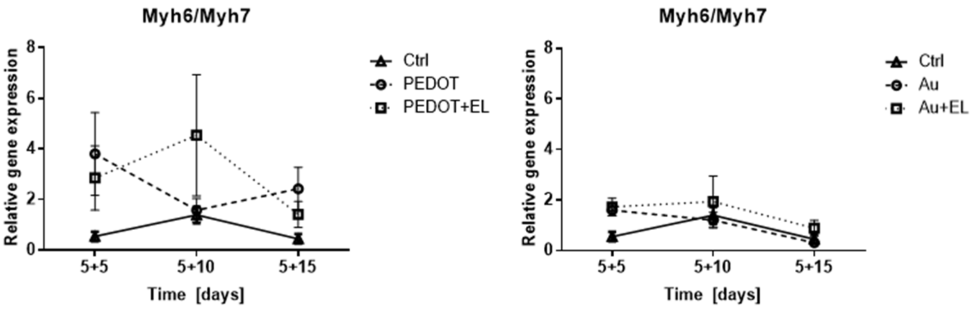

Figure S5. The ratios of Myh6 expression to Myh7 expression in cardiomyocytes on PEDOT:PSS based platform and platform with gold. The ratio of mRNA levels of Myh6 to Myh7was analyzed in mESCs line R1 which were differentiated for 20 days. Different time points were studied (5+5, 5+10,5+15d). This time point represents individual phases of differentiation. EBs adhered to platforms were treated on day 6 with square electric pulses (1 Hz, 200 mV/mm, pulse duration 100 ms) for 15 minutes (PEDOT+EL, Au+EL). Culture plastics (Ctrl) served as control, and comparison with platforms without electrostimulation (PEDOT, Au) was drawn. Data are expressed as mean ± SEM n ≥ 4). Differences between samples were analysed by paired t-test and considered statistically significant for $p < 0.05$; they are marked with asterisks for material vs. material + electrical stimulation and with hashtags for statistically significance to control.

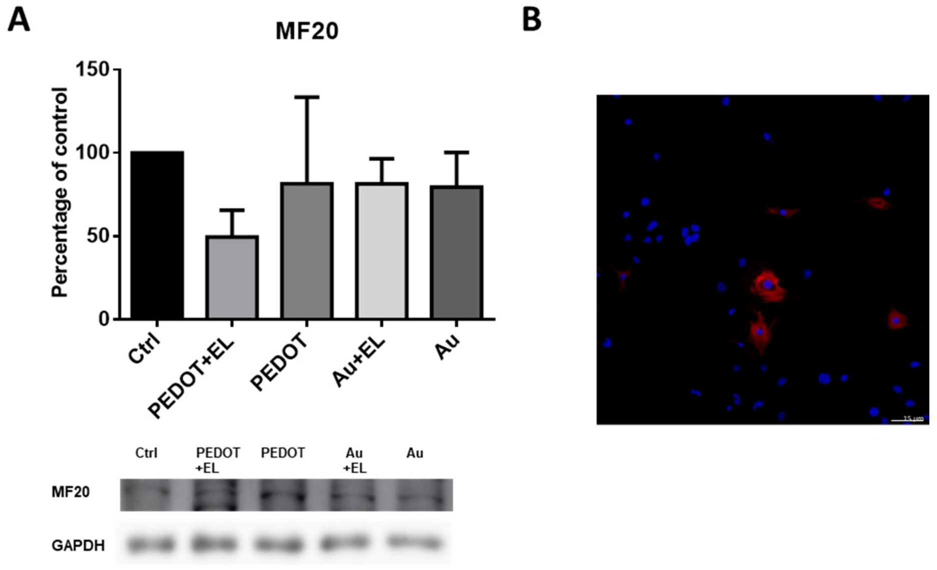

Figure S6. The expression of cardiac heavy myosin chains determined by MF20 antibody on PEDOT: PSS based platform and platform with gold.

The level of myosin heavy chain α and β (MHCα/β) was studied at the end-point (5+15d). EBs adhered to platforms were treated on day 6 with square electric pulses (EL) (1Hz, 200 mV/mm, pulse duration 100 ms) for 15 min (PEDOT+EL, Au+EL). Culture plastics (Ctrl) served as control, and comparison with platforms without electrostimulation (PEDOT, Au) was drawn. As a loading control, GAPDH was used. (A) Data from western blot are not robust enough. (B) In all variants, Immunofluorescence staining of embryoid clusters showed presence of myofibrils (red colour). Nuclei (blue) were stained with DAPI. The panel demonstrates typical observation in all variants. Data in (A) are expressed as mean ± SEM (n = 3). No statistically significant differences between samples were found by paired t-test $p < 0.05$.

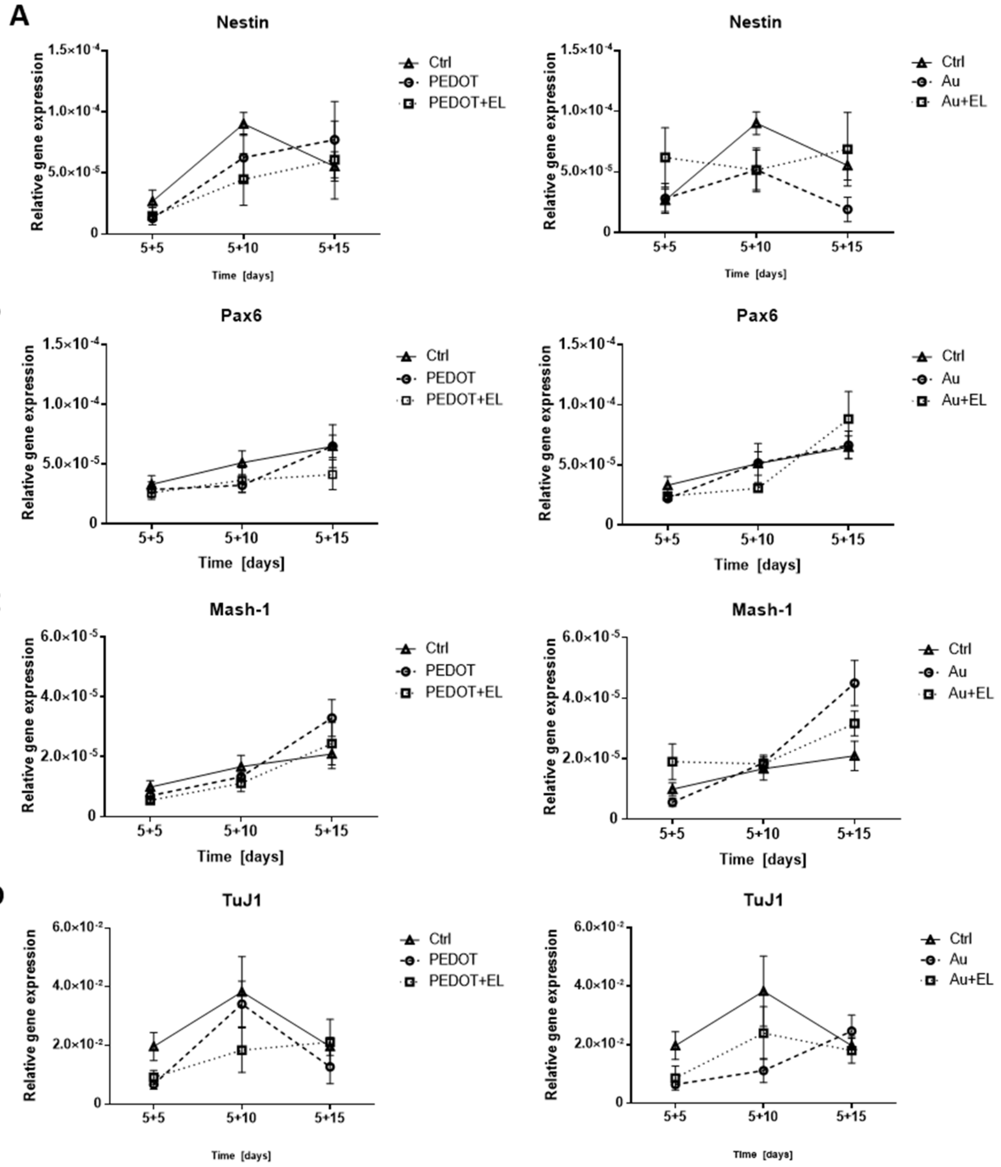

Figure S7. The effect of electrostimulation on expression cardiomyogenesis markers: Nestin (early neurogenesis), Pax6 (neural precursor), Mash-1 (neural maturation) and TuJ1 (neural maturation) on PEDOT: PSS based platform (left column) and the gold platform (right column). These genes represent markers of neural differentiation. The mRNA levels of Nestin, Pax6, Mash-1, TuJ1 were analyzed in mESCs line R1 which were differentiated for 20 days. Different time points were studied (5+5, 5+10,5+15d). This time point represents individual phases of differentiation. EBs adhered to platforms were treated on day 6 with square electric pulses (1 Hz, 200 mV/mm, pulse duration 100 ms) for 15 min (PEDOT+EL, Au+EL). Culture plastics (Ctrl) served as control, and

comparison to platforms without electrostimulation (PEDOT, Au) was drawn. Data are expressed as mean ± SEM ($n \geq 4$). Differences between samples were analyzed by paired t-test and considered statistically significant for $p < 0.05$; they are marked with asterisks for material vs. material + electrical stimulation and with hashtags for statistical significance to control.

Supplementary methods

Myosin heavy chain α and β (MHCα/β) detection by MF20 antibody

General steps of the western blotting were performed as described in the main paper. The mouse MF20 antibody was used (the hybridoma MF20, developed by Drs. Donald and Fischman, was obtained from the Developmental Studies Hybridoma Bank developed under the auspices of the National Institute of Child Health and Human Development and maintained by the University of Iowa, Department of Biological Sciences (Iowa City, IA, https://dshb.biology.uiowa.edu/MF-20, website accessed 20/05/2021), Corresponding secondary HRP-conjugated anti-mouse 1:2000 (Cell signaling, USA).

Myofibrils staining

For immunofluorescent staining were used cells 5+15d. These cells were dissociated with Accutase solution (Gibco, Thermo Fisher Scientific, USA) for 5 min at 37°C and plated on Falcon Culture Slides (Corning Incorporated; New York, NY, USA) coated with 0.1% gelatin. After 48 hours of incubation, the cells were washed in 1x phosphate buffered saline (PBS), fixed in 0.5% formaldehyde for 20 minutes at room temperature (RT), washed in 1x PBS, permeabilized with 0.1% TritonX-100 for 30 min at RT, and then twice washed in 1xPBS for 5 minutes. After 1 hour of blocking with 10% goat serum, the cells were incubated for 2h at RT with the primary antibody: mouse MF20 (MHCα/β; antibody detects both alpha and beta MHC isoforms). Several washing steps later, the cells were incubated for 1h at RT with DyLight 488 conjugate goat anti-mouse IgG (Thermo Fisher Scientific; Waltham, MA USA; TF35502). Nuclei were counterstained with DAPI (1 µg/ml). Cells were mounted on microscopic slides in Mowiol (Calbiochem; La Jolla, CA, USA) solution (10% Mowiol 4–88 prepared in 25% glycerol, 100 mM Tris–HCl, and 0.6% 1.4-diazabicyclo-[2.2.2]-octane, pH 8.5). Images were acquired using a confocal microscope (TCS SP5; Leica; Wetzlar, Germany) equipped with a 63x1.4 oil immersion objective.

Gene Expression Analysis

The expression of Nestin, Pax6 and Mash-1 was studied as described in the main paper. Primers and probes are indicated in Supplementary Table 1.

The expression of TuJ1 was determined using the DyNAmo HS SYBR Green qPCR Kit (Thermo Scientific™, Waltham, MA, USA) according to manufacturer instructions. TBP was taken as housekeeping gene. Primers and annealing temperatures are indicated in Supplementary Table 1.

Supplementary Table S1

Gene of Interest	Forward Primer 5′→3′	Reverse Primer 5′→3′	UPL Probe No.
Nestin	AGGCTTCTCTTGGCTTTCCT	AAGGGGGAAGAGAAGGATGTT	73
Pax6	TTGCATAAGAGGCATCGGCT	CAGCACCGAAAGCAAGACAC	78
Mash-1	AGGGATCCTACGACCCTCTTA	ACCAGTTGGTAAAGTCCAGCAG	6
			Annealing temperature
TuJ1	TGAGGCCTCCTCTCACAAGTA	GTCGGGCCTGAATAGGTGTC	62°C
TBP	ACCGTGAATCTTGGCTGTAAAC	GCAGCAAATCGCTTGGGATTA	60°C

Reference

1. Radaszkiewicz, K. A.; Sykorova, D.; Karas, P.; Kudova, J.; Kohut, L.; Bino, L.; Vecera, J.; Vitecek, J.; Kubala, L.; Pachernik, J. Simple non-invasive analysis of embryonic stem cell-derived cardiomyocytes beating in vitro. *Review of Scientific Instruments* **2016**, *87*, 024301.
